# Evaluation of Handheld Radionuclide Identifiers

**DOI:** 10.6028/jres.109.032

**Published:** 2004-08-01

**Authors:** L. Pibida, M. Unterweger, L. R. Karam

**Affiliations:** National Institute of Standards and Technology, Gaithersburg, MD 20899-8462

**Keywords:** ANSI N42.34, ANSI standards, gamma-ray detection, homeland security, isotope identifier, radionuclide identifier

## Abstract

Characterization of commercially available instruments for measurement and identification of unknown radionuclides was carried out in support of the development and testing of the American National Standards Institute (ANSI) standard, N42.34, “Performance Criteria for Hand-held Instruments for the Detection and Identification of Radionuclides.” Measurements were based on the performance of the devices, i.e., the capability of the detectors to ensure a correct radionuclide identification in a given time interval for various radioactive sources.

## 1. Introduction

A subset of commercially available hand-held radionuclide identifiers was acquired for characterization of their performance and accuracy of identification. The detector’s performance was evaluated against the radiological measurement requirements set forth in the draft American National Standards Institute (ANSI) standard, N42.34 “Performance Criteria for Hand-held Instruments for the Detection and Identification of Radionuclides.” As a results of this test as well as tests performed at Oak Ridge National Laboratory (ORNL) and Los Alamos National Laboratory (LANL) the standard was published as reference [1]. The greatest challenge for the devices in question is the need to correctly identify a wide variety of radionuclides in a specified and limited period of time. It is particularly difficult to design an identification algorithm that can analyze a broad range of radionuclides. Many factors such as acquisition time, calibration drift, temperature changes, changes in background radiation levels, etc., complicate this task. False positive and false negative identifications are common, particularly for spectra collected under realistically harsh field conditions. Furthermore, an instrument can identify only the nuclides it has been programmed to recognize. For practical reasons, the developers of instruments are forced to limit the number of radionuclides specified within the internal libraries for identification. Therefore, a number of failed or faulty identifications result from trying to measure a radionuclide that is not included in the library of a particular instrument.

## 2. Experimental Setup

### 2.1 Instruments Used

Four commercially available instruments were used in this evaluation. Three instruments are equipped with NaI(Tl) detectors, two of which have neutron detection capabilities and one instrument is equipped with a Cadmium-Zinc-Telluride (CZT). All of these detectors are available as commercially off-the-shelf (COTS) items. Some instruments self-calibrate each time the instrument is turned on, while others rely on the user’s decision to perform a specific calibration. Some instruments contain a calibration source to deliver a reference peak in the data, while others do not. Some use a fixed region-of-interest (ROI) method in their algorithm, while others try to fit the peaks and identify them. The general characteristics as stated by the manufacturers of all four detectors are listed in Table 1.

### 2.2 Sources Used

Table 2 shows the list of gamma-ray emitting radioactive sources used for the test. These sources are some of the sources listed in the ANSI N42.34 standard for testing this type of equipment. The sources used are calibrated NIST sources for which source activity values are given with an uncertainty of 0.5 % (for 1 σ).

### 2.3 Detectors Setup

Data from all of the detectors using a specific source was acquired simultaneously with the detectors set around the source on a horizontal table. The data for all four detectors was acquired for the same period of time (1 min of live time) and at the same source-to-detector distance (typically 15 cm). For weak sources such as ^57^Co, ^109^Cd, ^125^I, ^239^Pu, and Natural Uranium, the source-to-detector distance was reduced to 7.5 cm for all four detectors. For the detectors that do not have the ability to set a preset acquisition time, every effort was made to stop the acquisition after 1 min. It should be noted that the results do not necessarily represent the optimal performance of these instruments for identifying different radionuclides, as some of the detectors require a longer acquisition time or a smaller source to detector distance. A total of 445 measurements were made: 10 measurements per radionuclide per detector, with five additional measurements performed for one of the detectors to corroborate that positive identification was possible if given extra counting time. The measurements were repeated while placing a 2 mm stainless steel plate between the sources and the detectors. The background during the measurements was approximately 9 µR/h. During testing the ambient temperature varied between 20 °C and 22 °C and the relative humidity was 62 %.

### 2.4 Data Analysis

Correct identification of a given unknown radionuclide is a complex task. Five possible results defined in reference [2] were used for determination of the detectors performance.

The five possible results are defined as follows:

- “Correct” (C):
  The instrument correctly identified at least one most abundant isotope (MAI) present in the radioactive source as the isotope identified with the most confidence or with confidence less than only a minor daughter (see definition below), or in the case of background it means it identified either nothing or only ^40^K (which is naturally in the environment).
- “Conditionally correct" (CC):
  At least one MAI present was correctly identified, but with less confidence than something that was not present or could not be identified.
- “Minor daughter” (MD):
  A daughter or parent of a MAI was identified, but the instrument failed to identify a MAI or it identified a radionuclide known to be present with less than 10 % abundance. For example, identifying ^226^Ra when the source was ^238^U, or identifying the not-present natural grandparent ^232^Th when the source was ^228^Th.
- “False negative" (FN):
  The instrument gave no identification other than ^40^K (present in the background) when a radionuclide source was actually present. A false negative often meant that the spectrum has insufficient statistics for an identification to be made, or that the radionuclide was not in the instrument’s library.
- “False positive" (FP):
  The instrument identified one or more radionuclides that were not present as being present without making any correct identification other than ^40^K.

## 3. Results and Discussion

**Fig. 1:**
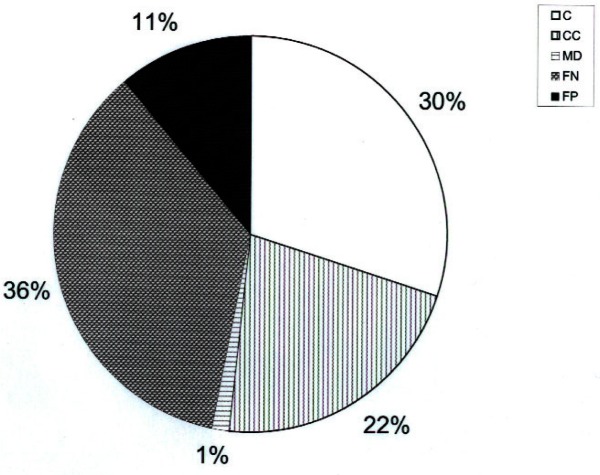
The percentage of correct (C), conditionally correct (CC), minor daughter (MD), false negative (FN) and false positive (FP) identification for all 445 measurements and all four detectors combined.

**Fig. 2:**
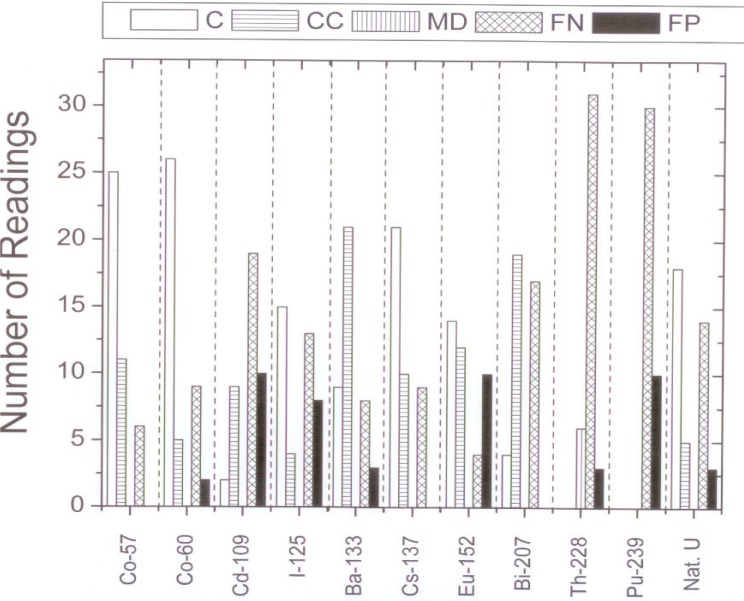
Combined number of readings from all four detectors for each category, defined as correct, conditionally correct, minor daughter, false negative and false positive, for all radioactive sources measured.

**Fig. 3:**
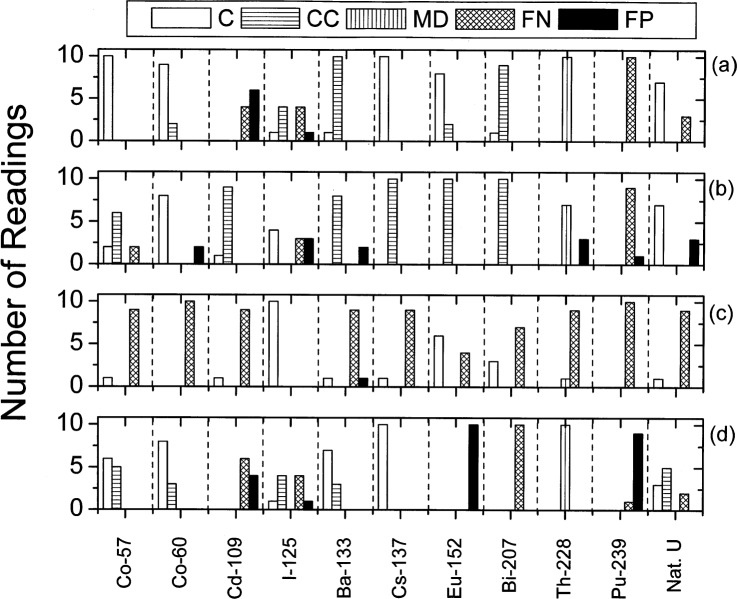
(a) readings for detector 1, (b) readings for detector 2, (c) readings for detector 3, (d) readings for detector 4 for each category; for all radioactive sources measured.

Figures 1 through 3 give the results of the measurements. Figure 1 represents the percentage of correct, conditionally correct, minor daughter, false negative and false positive identification for all 445 measurements. Figure 2 represents an aggregated performance of all four detectors to identify a particular radionuclide. Figure 3 shows the performance of each individual detector when identifying a given radionuclide.

The value of combining the results of all four detectors resides on the fact that presently users don’t have an extensive and detail summary of the individual performance of all commercially available instruments for all tests specified by the ANSI N42.34 standard. In this case the likelihood that a user will purchase any of these commercially available instruments is based on very few tests, so the combined data gives information about the overall performance of this type of instruments. Figure 1 shows that a correct identification was made for 30 % of the measurements; 22 % provided a conditionally correct identification, while a statistically insignificant 1.3 % provided minor daughter identification. In contrast, 36 % of the measurements gave a false negative, and 11 % a false positive, reading. For first responders, such as police, firefighters and customs agents, false negatives are of particular concern, as the instrument would fail to detect a source that is actually present, leading to an inappropriate or inadequate response to that source due to improper radionuclide identification. False positive and false negative determinations could, in principle, be solved rather easily through longer counting periods or more detailed spectrum analysis. Minor daughter identification might also present problems, but happens so rarely that it is of minimal concern.

From the breakdown of the results by individual radionuclide, as given in Fig. 2, we observe that the radionuclides that present the biggest challenge for identification are ^239^Pu, Natural Uranium, ^228^Th, ^207^Bi, ^109^Cd, and ^125^I. The main problem for identification of ^239^Pu, Natural Uranium, ^109^Cd, and ^125^I is their low gamma-ray energy for which background subtraction and absorption are of concern. For ^228^Th the problem is that the source is easily confused with ^232^Th, as both isotopes have similar gamma-ray lines. The problems with ^207^Bi might be due to the fact that it has several gamma lines, making it harder to identify properly.

In Fig. 3, the relative performance of all four individual detectors is given for comparison. All detectors had problems identifying ^239^Pu and ^228^Th. No defined trends were observed in the identification of all other radio-nuclides. Variations may come from differences within the library, source strength and/or detector efficiency. From Fig. 3 it can be observed that detectors 1 and 4 have a better overall performance. The figure also indicates that these two detectors perform relatively well when identifying radionuclides with one or two emission lines, but respond poorly to radionuclides emitting at more than two photon energies.

Detector 2 was observed not to give proper readings unless it was recalibrated every time the source was changed. Detector 4 had difficulties making a correct identification even when the distinct gamma-ray lines were in the spectrum, while detector 3 had difficulties making a correct identification in the one-minute acquisition time. These issues need to be taken into account before purchase, or during use, by the customer and should be addressed by the manufacturers.

When the measurements were repeated using a 2 mm stainless steel plate for shielding no correct identification was observed for all the sources used in the test by any of the detectors.

The ANSI standard N42.34 specifies that when identifying radionuclides, test results are considered acceptable when an instrument identifies the radionuclide(s) of interest, or that radionuclide(s) and expected daughter(s). It is considered not acceptable if the instrument identifies unexpected radionuclides or only the daughter(s) of the radionuclide(s) of interest. During testing the gamma dose rate at the detector from each source, unshielded or shielded, shall be 50 µR/h. The test shall consist of 10 trials for each radionuclide. The instrument shall be reset between each trial, if appropriate. The performance is acceptable when the instrument correctly identifies the radionuclide 8 out of 10 consecutive trials. The ANSI standard N42.34 specifications are appropriate for the use that this type of detectors will have in homeland security applications. Based on the results of the test, sub-classification of the identification results will be of no use to the user. Only a correct identification as specify by ANSI will be of real use when responding to a measurement in the field.

Uncertainty components are difficult to identify as the results come from the reading of a device and the result of the reading provides a correct, conditionally correct, minor daughter, false positive or false negative identification.

## 4. Conclusions

Initial characterization measurements on low to intermediate radioactivity sources of 11 individual radionuclides were performed with four commercially available handheld radionuclide identifiers. Even though the probability for a completely correct identification was only 30 %, the combined probability for proper “correct,” “conditional correct” and “minor daughter” classification of a source was approximately 53 %. That is of particular relevance for first responders, as their decision will be based mainly on the sum of these three probabilities. Nevertheless, 47 % of either negative or positive false identification should be considered as a significant negative benchmark of these devices, which must be kept in mind by a user interpreting any measurement, and which certainly requires optimization by the manufacturers. Further refined testing of handheld radionuclide identifiers and other commercially available equipment will contribute to characterizing the overall performance of these devices and improve testing procedures and device response. For example, environmental and field tests under realistic conditions, as indicated in ANSI N42.34, were not performed as part of this first test, but are critical to the overall performance characterization of these devices. This test served two purposes: the validation of the tests described in the draft ANSI N42.34 standard as well as the overall performance of radionuclide identifiers that are commercially available.

## Figures and Tables

**Table 1:** General characteristics of detectors tested

Detector number	Detector type	Detection range	Accuracy	Energy range (keV)
1	NaI(Tl)	10 nGy h^–1^ to Gy h^–1^	Not specified	20 to 2500
2	NaI(Tl)	Not specified	Not specified	30 to 3000
3	CZT	Not specified	Not specified	10 to 4000
4		8.78 nGy h^–1^	± 20 % (60 keV to 100 keV)	
	NaI(Tl)	8.78 mGy h^–1^	± 20 % (0.1 keV to 3 MeV)	50 to 3000

**Table 2:** List of radioactive sources

Radionuclides	Activity (Bq)	Main Gamma-ray energy lines (keV)
^57^Co	3 × 10^4^	122.06, 136.47
^60^Co	8 × 10^4^	1173.24, 1332.5
^109^Cd	3 × 10^4^	88.03
^125^I	2 × 10^4^	35.49
^133^Ba	4 × 10^4^	30.9, 80.99, 276.39, 302.85, 356.02, 383.85
^137^Cs	3 × 10^4^	661.66
^152^Eu	1 × 10^4^	121.78, 244.7, 344.28, 778.9, 1085.83, 1112.08, 1408.03
^207^Bi	2 × 10^4^	569.7, 1063.6, 1770.24
^228^Th	6 × 10^4^	238.63, 583.2, 727.18, 2614.6
^239^Pu	6 × 10^5^	16.4, 38.66, 51.62, 129.30
Natural U	5 × 10^5^	15.5, 143.76, 153.33, 185.72
